# BibliZap: An exploratory evaluation of an automated multi-level citation searching tool for systematic and rapid reviews

**DOI:** 10.1017/rsm.2026.10079

**Published:** 2026-03-24

**Authors:** Raphaël Bentegeac, Bastien Le Guellec, Victor Leblanc, Rémi Lenain, Luc Dauchet, Victoria Gauthier, Erwin Gerard, Emmanuel Chazard, Philippe Amouyel, Estelle Aymes, Aghilès Hamroun

**Affiliations:** 1 Public Health and Epidemiology, Lille University Hospital, France; 2 UMR1167 RID-AGE, Pasteur Institute of Lille, France; 3 Inserm, CHU Lille, U1172 LilNCog Lille Neuroscience & Cognition, Lille 2 University of Health and Law, France; 4 Neuroradiology, Lille University Hospital, France; 5 Hauts-de-France, Protection Maternelle et Infantile du Département du Nord, France; 6 Nephrology, Lille University Hospital, France; 7 CHU Lille, ULR 2694—METRICS, CERIM, Public Health Department, Lille 2 University of Health and Law, France

**Keywords:** automation, bibliographic search, citation searching, evidence synthesis, open-source tool, systematic review

## Abstract

The exponential growth of scientific literature poses increasing challenges for evidence synthesis. Systematic reviews (SRs) usually rely on keyword-based database searches, which are limited by inconsistent terminology and indexing delays. Citation searching—identifying studies that cite or are cited by known relevant articles—offers a complementary route to uncover additional evidence but remains poorly automated and integrated into screening workflows. We developed BibliZap, an open-source, fully automated citation-searching tool built on Lens.org data, performing multi-level forward and backward citation searches with relevance-based ranking. Its performance was evaluated across 66 published SRs, comparing five approaches: (1) PubMed-only searches; (2) PubMed followed by BibliZap restricted to the top 500 ranked results; (3) PubMed followed by full BibliZap screening; and (4–5) two exploratory early-stop strategies where BibliZap was initiated after identifying the first or the first three PubMed relevant records. The primary outcome was sensitivity, with secondary assessments of screening workload and precision. When used after PubMed screening, BibliZap increased mean sensitivity from 75% to 97%, achieving complete recall in over half of the reviews. Screening only the top 500 outputs still allowed over 90% of reviews to reach or exceed 80% recall. BibliZap recovered a median of three additional included articles per review, not retrieved by PubMed, while adding a median of 6,450 additional records. Citation searching via BibliZap enhances the completeness of evidence retrieval in SRs based on restricted database searches and supports transparent, scalable workflows adaptable to rapid and exploratory review contexts.

## Highlights

### What is already known?

Citation searching is increasingly recommended as a complementary strategy to database searches in systematic reviews (SRs). However, existing tools for citation searching are often limited by manual workflows, a lack of iterative capabilities, or opaque ranking mechanisms. To date, few studies have quantitatively assessed the performance of automated citation searching tools across a large and diverse set of reviews.

### What is new?

We present BibliZap, an open-source, fully automated citation searching engine capable of multi-level forward and backward citation searching with ranked output. Using a curated set of 66 published SRs, in which the set of included articles was manually extracted as a retrospective gold standard, we show that citation searching via BibliZap substantially improves sensitivity, recovering additional relevant studies compared with PubMed-only searches. We also compare performance across distinct search configurations and highlight the contribution of ranked prioritization for rapid reviews.

### Potential impact for journal readers

This study provides a robust, reproducible evaluation framework for citation searching tools and introduces a transparent and auditable solution for improving recall in evidence synthesis. BibliZap’s design and performance suggest that it may serve not only as a complement to traditional database queries but also as a standalone triage tool in exploratory or rapid reviews, offering a practical contribution to the methodology of evidence synthesis.

## 1 Introduction

The exponential growth of the scientific literature has transformed the way systematic reviews (SRs) are conducted. MEDLINE alone now indexes more than 35 million citations, with a publication volume doubling approximately every 17 years.^1^ As evidence-based medicine relies increasingly on comprehensive syntheses, SR teams must navigate a research landscape in which both information abundance and retrieval complexity have become structural challenges.^2^ ^–^ ^6^

The default approach to identifying relevant studies still relies on keyword-based queries in bibliographic databases such as PubMed.^7^ ^,^ ^8^ While indispensable, this strategy is known to suffer from sensitivity limitations in fields where articles are inconsistently indexed or terminology is heterogeneous and evolving.^9^ Even well-constructed queries often fail to retrieve all eligible records, prompting recommendations to supplement database screening with citation searching (i.e., identifying articles that cite or are cited by known relevant studies).^8^ ^,^ ^10^

Citation searching, often referred to as snowballing or citation mining/tracking, has become a recognized component of systematic search strategies.^10^ ^–^ ^14^ Forward and backward citation exploration can uncover relevant records that elude traditional database queries due to vocabulary mismatch or indexing lag.^15^ ^,^ ^16^ In a recent scoping review, Hirt et al. found that citation searching adds unique records in 96% of SRs in which it is used; however, as these were methodological studies focusing on cases where citation searching was applied, this figure likely overestimates its yield in unselected reviews.^10^ Based on this growing body of evidence, the recently published Terminology, Application, and Reporting of Citation Searching (TARCiS) statement outlines ten recommendations for the application and reporting of citation searching, including the possibility of conducting it over several rounds, whereby references identified at one step are used as new starting points for further exploration, and emphasizes the need for transparent documentation.^11^

Despite this consensus, citation searching remains underused and inconsistently applied.^17^ Its uptake is limited by the high time and resource burden required for a process whose yield is often heterogeneous and difficult to anticipate, due to incomplete database coverage, delayed indexing, and limited functionality for multi-seed citation export.^12^ ^,^ ^18^ To date, no free and fully automated tool has been available that enables multi-level citation searching with ranking and batch output across multiple seeds.^10^ ^,^ ^18^ More advanced platforms, such as Web of Science or Scopus, restrict automation or rely on proprietary relevance scores that are opaque and difficult to audit.^5^ In practice, these limitations severely hinder the integration of citation searching into time-sensitive or resource-limited SR workflows.^17^

To address the limitations of existing citation searching tools, we developed BibliZap, an open-source, fully automated engine that performs multi-level forward and backward citation searching and returns a ranked list of articles based on transparent relevance criteria. In this exploratory study, we evaluate BibliZap’s added value as a complementary tool for medical and health-related SRs. Using a retrospective gold standard derived from a previously selected corpus of published SRs,^19^ we assess its ability to identify relevant studies that were missed by PubMed-only searches. We examine its performance when applied at various time points in the review workflow, from early-stage integration (after identifying a few relevant articles) to post-database screening. We hypothesize that BibliZap, by operationalizing key recommendations from recent citation-searching guidelines,^11^ can substantially improve recall while maintaining a manageable screening workload.

## 2 Methods

### 2.1 Description of the BibliZap tool

BibliZap is an open-source, fully automated citation searching engine designed to support systematic, bidirectional citation searching with multi-level expansion. The tool is implemented in Rust, a modern, memory-safe programming language optimized for performance and reliability, enabling efficient processing of large citation networks. It leverages the Lens platform (https://www.lens.org), which aggregates citation data from CrossRef, OpenAlex, PubMed, PubMed Central, and CORE.^20^ ^,^ ^21^ As of early 2025, the Lens indexes over 272 million scholarly records, offering broad multidisciplinary coverage. In this study, only PubMed-indexed records accessible through the Lens were evaluated. Each run generates a reproducible log of all parameters, consistent with PRISMA-S reporting standards. BibliZap can be deployed through a web interface, an R package, or a command-line interface, depending on user preference. The codebase and documentation are publicly available on GitHub (https://github.com/BibliZap/biblizap-server).

**Figure 1:**
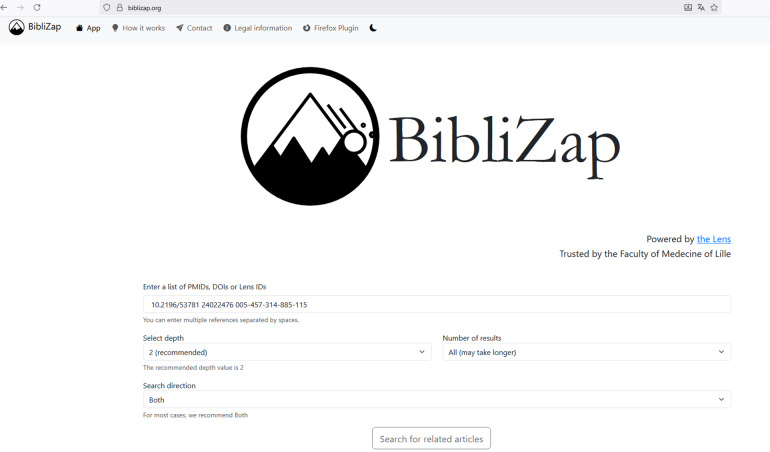
BibliZap web application input interface. User interface for launching a BibliZap query from the public web app (https://biblizap.org). Users input one or several seed identifiers (PMIDs, DOIs, or Lens IDs) separated by spaces, select the citation depth, direction (backward, forward, or both), and the number of results to retrieve. The application is powered by The Lens and includes a Firefox plugin for quick access from article pages. Figure 1 Long description.

To initiate a query, users input one or several identifiers (PMID, DOI, or Lens ID) as seed references. BibliZap then performs a recursive traversal of the citation graph, retrieving articles that cite or are cited by the input set (forward and backward citation searching^13^). In practice, BibliZap enables a multi-level expansion of the citation graph, in which all records retrieved at a given level are reused as seeds for the next traversal step.^13^ Each additional level increases the number of API requests and retrieved records by roughly two orders of magnitude, which makes the default depth of 2 a pragmatic trade-off between completeness and computational feasibility. Expansion up to depth 3 is technically supported, though not recommended for routine use. Each article in the corpus receives a ‘relevance score’, defined as the sum of its occurrences across all citation paths:

$$\begin{array}{r} {\mathrm{Relevance}\;\mathrm{score}}_{\mathrm{article}}=\sum_{i=1}^{d}A_{i}, \end{array}$$

where *A_i_* is the number of times the article appears at depth *i*, and *d* is the selected depth (Figure 1). When the search is limited to a single direction—either forward or backward—depth-2 expansion retrieves specific subsets of the citation network. A forward-only configuration identifies papers citing those that already cite the seed articles, while a backward-only configuration retrieves papers cited by those already referenced by the seeds. When both directions are selected, these outputs naturally encompass co-cited and co-citing records, corresponding to indirect relationships within the second-level graph expansion.

### 2.2 Gold-standard corpus

To evaluate BibliZap’s performance, we used a curated set of SRs previously assembled by our team.^19^ These reviews were published between 2012 and 2021 in six high-impact general medicine journals (The New England Journal of Medicine, The Lancet, JAMA, BMJ, Annals of Internal Medicine, and Nature Reviews Disease Primers). Eligible SRs had to (i) provide a reproducible PubMed search query, (ii) be fully accessible on PubMed Central, and (iii) explicitly list the studies included in the final evidence synthesis.

For each SR, the gold standard was defined as the set of included articles contributing to the review’s results or meta-analyses. This set was manually extracted by four reviewers (VL, RB, BLG, and AH) from the main text and supplementary materials. References used for background discussion, general epidemiology, methodological justifications, or reporting standards (e.g., PRISMA, GRADE) were excluded. Only studies with a PubMed ID were retained, enabling direct comparison between PubMed queries and BibliZap outputs. The proportion of excluded articles without a PMID was below 5%.

### 2.3 Search strategies

We evaluated five search strategies to identify the set of included studies (gold standard). Approaches 1–3 were designed to assess citation searching as a complement to full SR workflows, while Approaches 4 and 5 simulated rapid or focused searching scenarios using early stopping based on PubMed’s Best Match ranking.^22^ Best Match is PubMed’s default relevance-based algorithm, which ranks records using a machine learning model that prioritizes term frequency, citation patterns, and recency.

- **Approach 1 (PubMed)**: Full manual screening of all records retrieved by the original PubMed query (no BibliZap).
- **Approach 2 (PubMed + BibliZap top 500)**: After full PubMed screening, BibliZap was seeded with all gold-standard articles retrieved by PubMed, and only the 500 highest-ranked BibliZap results were screened.
- **Approach 3 (PubMed + BibliZap)**: After full PubMed screening, BibliZap was seeded with all gold-standard articles retrieved by PubMed, and the complete ranked output was screened in full.
- **Approach 4 (PubMed early stop + BibliZap)**: PubMed results were screened in Best Match order, and the process was stopped after identifying the first gold-standard article. This record was used as the sole seed for BibliZap, whose output was then screened in full.
- **Approach 5 (PubMed late stop + BibliZap)**: PubMed results were screened in Best Match order until three gold-standard articles were identified, which were then used jointly as seeds for BibliZap, whose output was then screened in full.

In all BibliZap-related configurations (Approaches 2–5), the following parameters were standardized: bidirectional search, depth of 2 levels, and a maximum output size of 10,000 articles, consistent with the retrieval limit imposed by the PubMed search engine. For each run, the SR itself was excluded from the citation graph to avoid circular chaining and unfairly improving Biblizap’s performance. Additionally, to match the original review’s publication context, a time filter was applied to restrict results to articles published during the SR’s publication window.

### 2.4 Metrics and statistical analysis

The primary outcome was sensitivity, defined as the number of gold standard articles retrieved by each approach divided by the total number of articles in the gold standard set. For each strategy, we estimated (i) the sensitivity across all included reviews, (ii) the proportion of reviews reaching predefined sensitivity thresholds (≥80%, ≥90%, and 100%)—with 90% often cited in the methodological literature as a commonly targeted recall level in SRs (although acceptable thresholds remain context-dependent),^23^ ^,^ ^24^ and 80% a pragmatic threshold for exploratory or rapid review contexts^25^—(iii) the number of articles screened, and (iv) precision, defined as the proportion of included gold-standard articles among all screened records. All performance metrics were summarized as mean values with 95% confidence intervals estimated by bootstrap resampling. Cumulative recall curves (with 95% confidence interval) were plotted to represent the proportion of retrieved gold standard articles as a function of the number of articles screened. PubMed results were analyzed in BestMatch order; BibliZap results were ranked by descending relevance score.

All statistical analyses were performed using R (version 4.2.0) and Python. Data wrangling and visualization were performed using the dplyr and ggplot2 packages (R).

## 3 Results

### 3.1 BibliZap interface and workflow

**Figure 2:**
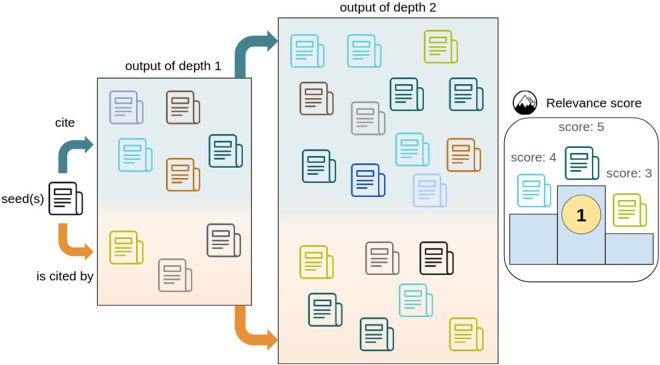
Overview of BibliZap’s citation searching process. Starting from one or more seed articles (bottom left), BibliZap performs forward (blue arrow: articles citing the seed) and backward (orange arrow: articles cited by the seed) citation exploration. The first expansion level (Depth 1) retrieves all directly linked articles. These are then used as new seeds to perform a second round of forward and backward citation searching (Depth 2), further expanding the network. In the figure, articles are color-coded to allow visual identification of their propagation through the network. The final output includes all unique articles retrieved across both expansion levels. Each article is assigned a relevance score, defined by the number of times it appears across all citation paths. As illustrated in the right panel, articles that appear most frequently receive the highest scores and are ranked accordingly, enabling transparent prioritization for screening.

**Figure 3:**
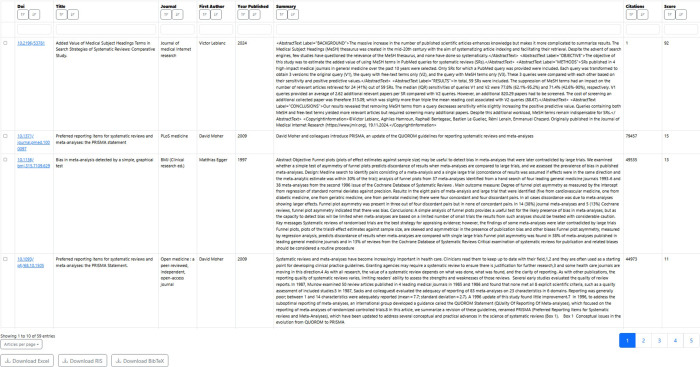
Example of BibliZap output interface. Screenshot of the web application output table after running a citation query. Retrieved articles are ranked by a cumulative relevance score and presented with their metadata, including DOI, title, journal, first author, publication year, summary, citation count, and score. Users can filter, sort, and download results for further screening or integration into reference management tools. Figure 3 Long description.

BibliZap can be accessed via a user-friendly web interface (https://biblizap.org, Figure 2), through an R package for local deployment, or via a dedicated Firefox browser extension. Users can perform a multi-seed query by simply separating each identifier (PMID, DOI, Lens ID, or a mix) with a space (Figure 2). Results are returned as a structured table sorted by decreasing score, and include the following fields: DOI, Title, Journal, First Author, Year Published, Summary, Number of Citations, and Relevance Score (Figure 3). A companion log file details the parameters used in each run, ensuring full reproducibility and alignment with PRISMA-S reporting standards.^26^ Export options include both spreadsheet (.xlsx) and RIS formats, ensuring compatibility with a wide range of reference management software. Users can choose to export up to 10,000 ranked records or the entire retrieved corpus at any configured search depth.

### 3.2 Overall sensitivity and screening burden

Among the 70 SRs initially included, 66 were retained for analysis. In the remaining four cases, the original PubMed search query provided by the authors retrieved no results when re-executed in the PubMed search engine. The final set of 66 reviews represented a total corpus of 2,675 included single references.

Using PubMed only (Approach 1), the mean sensitivity was 75% (95% CI 68–81) with a median of 1,889 articles screened (range 27–10,000) and a mean precision of 2.1% (95% CI 1.5–2.7). With the addition of BibliZap restricted to the top 500 ranked results (Approach 2), sensitivity increased to 91% (95% CI 88–94) with a median of 2,388 articles screened (range 527–10,500) and a mean precision of 1.5% (95% CI 1.1–1.9). When the full-ranked BibliZap output was screened (Approach 3), mean sensitivity reached 97% (95% CI 95–98) with a median of 8,422 articles (range 2,110–19,168) and a mean precision of 0.4% (95% CI 0.3–0.5). The early-stop configuration, where PubMed screening was halted after identifying the first relevant article (Approach 4), achieved a sensitivity of 75% (95% CI 68–81) with 6,126 articles screened (range 0–9,999) and a mean precision of 0.5% (95% CI 0.4–0.7). Finally, the late-stop configuration based on the first three PubMed-identified articles (Approach 5) reached 90% (95% CI 86–93) with a median of 7,198 articles (range 3,305–9,623) and a mean precision of 0.5% (95% CI 0.4–0.6).

In the full BibliZap configuration (Approach 3), the number of additional articles retrieved and screened from BibliZap after PubMed ranged from 2,083 to 9,168 (median: 6,450). Among these, the number of additional gold-standard articles recovered ranged from 1 to 78 per review (median: 3). When restricted to the top 500 ranked outputs, citation searching with BibliZap retrieved a median of two additional included articles per review (range: 0–55). Detailed distributions of recall, precision, and screening volume for each approach—and for each of the 66 SRs individually—are presented in Supplementary Table 1.

**Figure 4:**
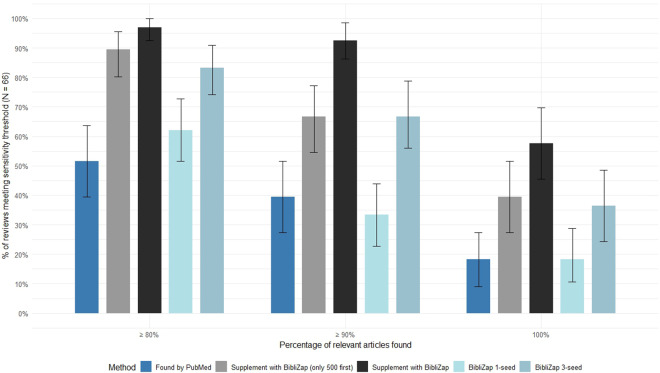
Proportion of relevant articles retrieved per systematic review, according to the three main citation searching configurations. Each horizontal bar represents one review (N = 66). Stacked segments indicate the proportion of included (gold-standard) articles retrieved using PubMed only (Approach 1, dark blue), PubMed supplemented with the top 500 BibliZap results (Approach 2, grey), and PubMed supplemented with the full BibliZap output (Approach 3, black). Bars are ordered by overall sensitivity within each review. Figure 4 Long description.

**Figure 5:**
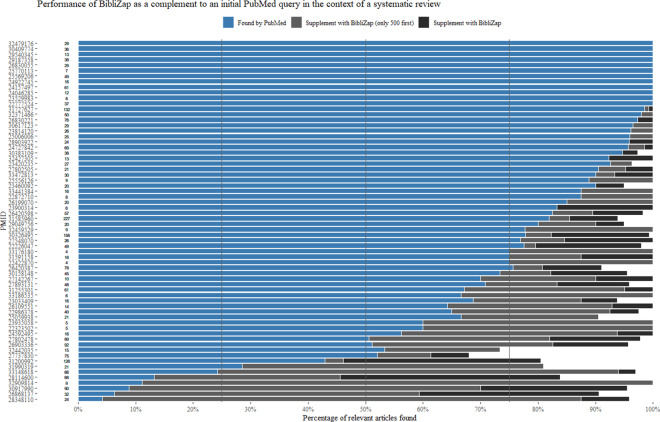
Proportion of systematic reviews (N = 66) meeting predefined sensitivity thresholds (≥80%, ≥90%, and 100%) across the five search strategies. Bars represent mean proportions with 95% confidence intervals. Approaches include: PubMed only (Approach 1, dark blue), PubMed + BibliZap top 500 (Approach 2, grey), PubMed + BibliZap full output (Approach 3, black), PubMed early stop + BibliZap (Approach 4, light blue), and PubMed late stop + BibliZap (Approach 5, teal). Figure 5 Long description.

Among the 30 reviews for which PubMed retrieved fewer than 90% of the included articles, supplementation with the full BibliZap output increased recall above this threshold in 22 cases. Similarly, of the 18 reviews with sensitivity below 80% using PubMed alone, 17 surpassed this threshold after applying full BibliZap citation searching. These review-level distributions of recall, stratified by approach and threshold, are illustrated by horizontal stacked bars (Figure 4). At the aggregated level, a sensitivity of ≥80% was reached in 52% of reviews using PubMed only, in 89% of reviews using the PubMed + top 500 BibliZap-ranked articles (Approach 2), and in 97% using the PubMed + full BibliZap output (Approach 3). The proportions of reviews reaching ≥90% sensitivity were 39%, 67%, and 92%, respectively. Complete recall (100%) was achieved in 18% of reviews with PubMed alone, 39% with top-500 BibliZap supplementation, and 58% with full BibliZap output screening (Figure 5).

### 3.3 Cumulative recall

**Figure 6:**
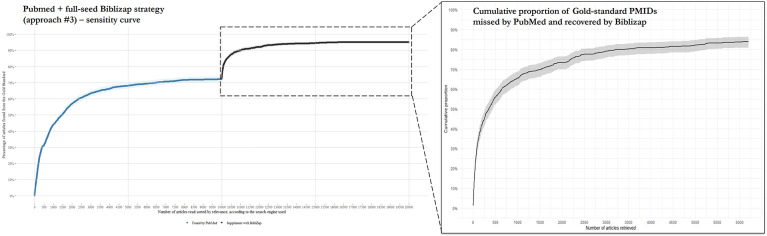
Cumulative recall curve for the PubMed + full-seed BibliZap strategy (Approach 3). Cumulative sensitivity is plotted as a function of the number of articles screened, in descending relevance order (Best Match for PubMed; BibliZap relevance score for citation searching). The transition between PubMed (dark blue) and BibliZap (black) corresponds to the point where PubMed screening ends and citation searching begins. Right panel: among gold-standard articles not retrieved by PubMed (Approach 1), the cumulative proportion recovered by BibliZap (Approach 3) is plotted against the number of ranked articles screened. Shaded areas represent 95% confidence intervals. Figure 6 Long description.

At the aggregated level, sensitivity reached 75% (95% CI: 73.3%–76.6%) after full screening of PubMed records and increased to 90% (95% CI: 88.8%–91.1%) after screening 3,000 additional articles ranked by BibliZap (Figure 6). The curve gradually plateaued near complete recall at around 9,000 BibliZap-ranked articles screened, reaching 96.6% sensitivity (95% CI: 94.3%–97.6%). Focusing on the subset of gold-standard articles missed by PubMed but recovered through BibliZap, 56% were found within the top 500 ranked results (95% CI: 52.3%–59.6%), 66% within the top 1,000 (95% CI: 61.8%–68.8%), 73% within 2,000 (95% CI: 69.9%–76.5%), and 84% within the top 6,000 (95% CI: 80.9%–86.3%). These cumulative detection rates for PubMed-missed articles are also shown in Figure 6 (inset).

## 4 Discussion

This study assessed the effectiveness of BibliZap, an open-source citation searching engine, in enhancing the comprehensiveness of SR searches beyond conventional PubMed queries. Our findings demonstrate that supplementing standard PubMed searches with multi-level citation searching substantially improves recall, increasing average sensitivity from 75% to 97%. BibliZap proved particularly effective at identifying relevant articles missed by PubMed searches, retrieving approximately 73% of these missed records within the first 2,000 ranked results. The prioritization and ranked outputs provided by BibliZap thus enabled an efficient recovery of crucial articles while maintaining a manageable screening workload. These results underscore the practical value of automated citation searching methods and highlight BibliZap’s potential to operationalize current methodological recommendations for systematic literature searching.

These findings align with and extend prior evidence showing that citation searching can enhance recall when supplementing database searches, here demonstrated specifically in the context of PubMed-only queries.^10^ ^,^ ^15^ ^,^ ^16^ ^,^ ^27^ Only a limited number of studies have quantified this added value. Haddaway et al. (2022), in an evaluation of the Citationchaser tool across ten SRs, reported that backward citation searching alone recovered 14% of included records that had not been identified through traditional database queries.^18^ In contrast, Wright et al. (2014), examining forward citation chasing across 40 Cochrane reviews, found that only one additional included study was identified, underscoring the variable yield of citation-based strategies depending on topic and method.^28^ Beyond such isolated estimates, Hirt et al. (2023) provided a scoping synthesis of 47 reviews that used citation chasing and concluded that backward or forward strategies yielded unique records in over 90% of cases.^10^ Similarly, Frandsen and Eriksen (2023) showed that supplementary search techniques, including citation searching, consistently retrieved additional eligible studies in qualitative SRs, reinforcing the importance of systematically integrating these methods rather than treating them as optional add-ons.^29^ The diversity and heterogeneity of citation coverage across databases were further highlighted by Gusenbauer (2024), who compared 59 citation indexes and documented substantial differences between platforms such as Lens.org, OpenAlex, Scopus, and Web of Science.^27^ In this context, our results add to the evidence base by providing a large-scale, quantitative evaluation of an open-source tool—BibliZap—implementing a fully automated, multi-seed, multi-level citation-searching process with ranked outputs built on the Lens citation graph.

Among currently available tools, *Citationchaser* is the most comparable open-source solution.^18^ It supports backward and forward citation retrieval and allows multiple inputs, but is limited to one level of expansion and does not provide ranking or relevance-based prioritization. While useful for manual citation exploration, its output must be reviewed in full, which may be impractical for large citation neighborhoods. Other tools, such as *CoCites*, recommend related papers based on co-citation and bibliographic coupling but rely on a proprietary algorithm and do not generate reproducible, parameterized outputs.^30^ *Scite* provides unique features by identifying citation intent (e.g., supporting or contrasting), but it is not designed for high-recall evidence retrieval.^31^ *Citation Cloud*, integrated with the Anne O’Tate interface for PubMed, offers graphical access to an article’s citation neighborhood, including co-cited and bibliographically coupled works.^32^ However, it is centered on a single article, does not support multi-seed workflows, and lacks batch screening functionality. Other recent tools, such as *SpiderCite* and *Paperfetcher*, further illustrate the diversity of available solutions.^27^ Both perform single-level citation expansion and lack ranking or parameterized output. *Paperfetcher* allows either forward or backward exploration through a lightweight interface, whereas *SpiderCite* focuses on one-level citation retrieval. In contrast, BibliZap was designed to align with several key recommendations of the TARCiS statement.^11^ Its recursive traversal corresponds to multi-level expansion of the citation network rather than screening-based iteration. This design choice allows comprehensive graph exploration while maintaining a transparent and parameterized structure. It supports bidirectional citation searching (Recommendation 2), accepts multiple seed records (Recommendation 4), and allows multi-level expansion (Recommendation 8). While BibliZap does not yet implement automated de-duplication as outlined in Recommendation 7, this functionality is under active development. The tool’s open-source architecture contributes to its transparency and long-term sustainability, consistent with Recommendation 10.

Moreover, although prioritization of results is not yet formally included in the TARCiS recommendations, it has been identified as a key research priority.^11^ BibliZap’s ranking algorithm directly contributes to this agenda by supporting early stopping strategies, which may be particularly valuable in time-constrained or resource-limited contexts.^10^ ^,^ ^18^ In our study, more than half of the relevant studies missed by PubMed were retrieved within the top 500 BibliZap-ranked articles, and over 70% within the first 2,000, highlighting the system’s ability to concentrate relevant literature at the top of the output. This capacity to prioritize high-yield records early in the screening process echoes prior methodological reflections that acknowledge the potential of standalone citation searching outside the traditional SR framework, particularly in scoping reviews or exploratory syntheses.^10^ Such approaches are especially relevant when conventional keyword-based retrieval may fail to identify all pertinent studies. Our results further show that a lightweight configuration (triggering BibliZap after the identification of only three relevant articles in PubMed) yielded a mean sensitivity of 90%, at the cost of a significant increase in workload. This supports, however, the idea that citation searching, when supported by a ranking algorithm, can offer flexible entry points into the literature, depending on user needs and available resources. This perspective aligns with earlier empirical work by Belter (2016), who demonstrated through empirical analysis of eight bibliometric reviews that forward citation searching alone retrieved between 56% and 88% of relevant studies, with a median recall of 72%, and with his subsequent study (2017), which introduced a citation-ranking algorithm and achieved up to 87% recall across 23 SRs.^33^ ^,^ ^34^ His work highlights the potential of citation networks to serve as primary data sources in structured but non-systematic evidence syntheses. BibliZap’s ability to deliver multi-level, ranked, and transparent outputs from multiple seeds thus makes it particularly suited for such alternative applications, offering a flexible, auditable, and efficient citation searching solution beyond its role in formal SRs.

This exploratory study has several limitations. First, it was conducted retrospectively using published SRs. Although these reviews spanned diverse medical topics and were selected from high-impact journals, prospective validation within real-time screening workflows would provide a more robust assessment of usability in practice. Second, we defined the gold standard as the set of articles included in each review. These inclusion sets may be incomplete or imprecise, particularly when the reporting of methods or supplementary materials was suboptimal. Nonetheless, all gold-standard articles were manually verified by independent reviewers to ensure fidelity, and we excluded records not indexed in PubMed to allow consistent comparison across tools. Third, our evaluation was limited to the PubMed component of each review, meaning that ‘missed’ refer specifically to records not retrieved by PubMed. While this does not capture the multi-database strategies used in most SRs, fewer than 5% of included studies in our corpus lacked a PubMed ID, suggesting limited impact on overall findings. This restriction does not constrain the applicability of BibliZap, which can be seeded from any identifier indexed by Lens.org. Fourth, BibliZap currently performs direct citation searching and, at depth-2 expansion, inherently retrieves co-cited and co-citing records. However, it does not yet implement dedicated co-citation or bibliographic-coupling algorithms as standalone search strategies,^35^ which could represent a future enhancement of its functionality. Although this design choice promotes transparency by ensuring that each connection is grounded in an explicit citation link, it may limit the discovery of conceptually related but citation-distant literature. At present, the actual impact of co-citation and related methods on systematic retrieval remains poorly evaluated, and their theoretical appeal requires further empirical validation.^10^ Citation-based approaches, including BibliZap, may also underrepresent very recent or sparsely cited studies, and their ranked outputs cannot mitigate publication bias—a limitation previously emphasized by Belter,^33^ who noted that certain study types are rarely identifiable through citation relationships alone. However, because BibliZap’s ranking integrates both forward and backward links rather than raw citation counts, it can still highlight recent or methodologically comprehensive articles that cite relevant prior work, partially attenuating this limitation. Moreover, we did not formally compare depth-1 and depth-2 configurations. While depth 2 was selected as a pragmatic default balancing recall and feasibility, future work should directly assess the marginal benefit and workload implications of single- versus two-level expansion using BibliZap. Finally, our evaluation focused exclusively on sensitivity and screening burden; we did not assess how the use of BibliZap might influence the overall conclusions or strength of evidence in the resulting SRs.

Its compatibility with scripting environments and screening software makes BibliZap particularly well-suited for integration into semi-automated evidence synthesis pipelines. Beyond traditional use cases, its structured and ranked outputs provide a compelling foundation for downstream coupling with large language models (LLMs) trained to support citation screening.^36^ ^,^ ^37^ Recent findings by Tran et al. suggest that LLMs can assist in automating early phases of study selection by classifying abstracts with reasonable reliability.^38^ In this context, BibliZap could act as an upstream filtering mechanism, delivering pre-prioritized corpora that reduce the burden on LLM triage models and improve overall pipeline efficiency. Such synergy is not only a matter of time savings, but it may also improve accuracy. Citation screening is a known bottleneck in SRs, where human reviewers have been shown to miss up to 11% of relevant studies during initial selection.^39^ ^,^ ^40^ By combining BibliZap’s iterative citation searching with LLM-based classification, future systems could mitigate human error while accelerating throughput, particularly in rapid or living reviews, where screening steps must be repeated regularly. Further developments could also explore user-defined weighting schemes within the ranking algorithm (e.g., emphasizing recency, citation context, or specific metadata fields), advanced filtering capabilities (e.g., excluding editorials or protocols), or native integration with review management platforms such as Covidence or Rayyan.^41^ Coupled with transparent logging of parameters and open access to its underlying codebase, these advances would support the development of fully reproducible, auditable, and scalable workflows for modern evidence synthesis.

To conclude, BibliZap is a transparent, configurable, and automated citation searching tool that improves sensitivity in SRs conducted on restricted database searches (here, PubMed). Its feasibility is best demonstrated in rapid or resource-limited review contexts, particularly when screening is limited to the top-ranked outputs. BibliZap operationalizes key recommendations from the TARCiS statement and offers practical value for both exhaustive and exploratory evidence retrieval tasks.

## Supporting information

10.1017/rsm.2026.10079.sm001 Bentegeac et al. supplementary material Bentegeac et al. supplementary material

## Data Availability

The dataset of 66 SRs used in this study is available from the corresponding author upon reasonable request. This includes the original PubMed search queries, manually curated gold-standard article lists, and extracted metadata used for performance evaluation. In addition, all source code and documentation required to run BibliZap are freely available on GitHub (https://github.com/BibliZap/biblizap-server), including: (i) the R package and command-line interface; (ii) instructions for replicating all analyses and figures from this paper; (iii) example seed inputs and configuration files; (iv) tools for exporting and formatting results; and (v) the complete backend and frontend code. This ensures full reproducibility and transparency of our evaluation pipeline.

## Supplementary material

To view supplementary material for this article, please visit http://doi.org/10.1017/rsm.2026.10079.

## Long descriptions

### Figure 1 Long description

The interface is organized into three main vertical sections.

At the top, a navigation bar includes the BibliZap logo, links for App, How it works, Contact, Legal information, Firefox Plugin, and a dark mode toggle. Below this is a large central logo featuring a mountain and a falling meteor next to the text BibliZap. To the right, text reads Powered by the Lens and Trusted by the Faculty of Medecine of Lille.

The middle section contains the search form. A text field labeled Enter a list of P M I D s, D O I s or Lens I D s contains example identifiers separated by spaces. Below this are two dropdown menus. The left menu, Select depth, is set to 2 recommended. The right menu, Number of results, is set to All may take longer. Underneath these is a wide dropdown menu for Search direction set to Both.

At the bottom center is a large button labeled Search for related articles.

Navigate back to Figure 1.

### Figure 3 Long description

The interface features a structured table with nine columns. From left to right, the headers are: D O I, Title, Journal, First Author, Year Published, Summary, Citations, and Score. Each header includes filter and sort icons.

Below the headers, the table displays four visible rows of research articles.

- The first row shows a 2024 article by Victor Leblanc in the Journal of medical internet research with a score of 92.

- The second row lists the P R I S M A statement by David Moher from 2009 in P L o S medicine with 79,457 citations.

- The third row features a 1997 article on bias in meta-analysis by Matthias Egger in B M J.

- The fourth row is another entry for the P R I S M A statement from 2009 in Open medicine.

The Summary column contains dense blocks of text including abstract labels like BACKGROUND and METHODS. At the bottom left, a status message indicates Showing 1 to 10 of 59 entries. The bottom right contains pagination buttons numbered 1 through 5. Three download buttons are located at the very bottom: Download Excel, Download R I S, and Download BibTeX.

Navigate back to Figure 3.

### Figure 4 Long description

The Y axis represents the percentage of reviews meeting the sensitivity threshold N equals 66, ranging from 0 percent to 100 percent. The X axis categorizes results by the percentage of relevant articles found at three thresholds: greater than or equal to 80 percent, greater than or equal to 90 percent, and 100 percent. Each threshold contains five bars with vertical error bars.

At the greater than or equal to 80 percent threshold:

* Found by PubMed (dark blue): approximately 52 percent.

* Supplement with BibliZap only 500 first (grey): approximately 90 percent.

* Supplement with BibliZap (black): approximately 97 percent.

* BibliZap 1-seed (light blue): approximately 62 percent.

* BibliZap 3-seed (medium blue): approximately 83 percent.

At the greater than or equal to 90 percent threshold:

* Found by PubMed: approximately 40 percent.

* Supplement with BibliZap only 500 first: approximately 67 percent.

* Supplement with BibliZap: approximately 93 percent.

* BibliZap 1-seed: approximately 33 percent.

* BibliZap 3-seed: approximately 67 percent.

At the 100 percent threshold:

* Found by PubMed: approximately 18 percent.

* Supplement with BibliZap only 500 first: approximately 40 percent.

* Supplement with BibliZap: approximately 58 percent.

* BibliZap 1-seed: approximately 18 percent.

* BibliZap 3-seed: approximately 37 percent.

Across all thresholds, the Supplement with BibliZap method (black bar) consistently achieves the highest percentage of reviews meeting the sensitivity target.

Navigate back to Figure 4.

### Figure 5 Long description

A horizontal stacked bar chart titled Performance of BibliZap as a complement to an initial PubMed query in the context of a systematic review.

Axes and Legend

* The Y-axis lists 66 unique P M I D identifiers with associated sample sizes.

* The X-axis represents the Percentage of relevant articles found, ranging from 0 percent to 100 percent in 10 percent increments.

* The legend identifies three categories: Found by PubMed (blue), Supplement with BibliZap only 500 first (grey), and Supplement with BibliZap (black).

Data Trends

* The bars are ordered from highest PubMed sensitivity at the top to lowest at the bottom.

* Top 15 rows: PubMed alone achieves 100 percent sensitivity, represented by solid blue bars extending the full width of the chart.

* Middle rows: PubMed sensitivity gradually decreases from approximately 95 percent to 70 percent. Grey and black segments appear on the right, showing that BibliZap supplements the remaining 5 percent to 30 percent to reach or approach 100 percent total sensitivity.

* Bottom 10 rows: PubMed sensitivity drops significantly, reaching as low as approximately 5 percent for P M I D 28348110. In these cases, the grey (top 500) and black (full output) BibliZap supplements account for the majority of the relevant articles found, often bringing the total sensitivity above 90 percent.

Navigate back to Figure 5.

### Figure 6 Long description

The figure consists of two panels.

Left Panel: PubMed plus full-seed BibliZap strategy approach 3 sensitivity curve.

* The Y-axis represents the percentage of articles found from the Gold Standard, ranging from 0 percent to 100 percent.

* The X-axis represents the number of articles read sorted by relevance, ranging from 0 to 20,000.

* The data begins with a dark blue line representing articles found by PubMed. It shows a steep logarithmic increase, reaching approximately 70 percent recall at 3,000 articles, then plateaus slightly until 10,000 articles.

* At the 10,000 mark, a vertical dashed box indicates the transition to a black line representing the supplement with BibliZap. This line shows a sharp vertical jump from 72 percent to approximately 88 percent, then continues to rise asymptotically toward 95 percent recall as it reaches 20,000 articles.

Right Panel: Cumulative proportion of Gold-standard P M I D s missed by PubMed and recovered by BibliZap.

* The Y-axis represents cumulative proportion from 0 percent to 100 percent.

* The X-axis represents the number of articles retrieved from 0 to 6,000.

* A black line shows a rapid initial increase, reaching 50 percent recovery within the first 500 articles. The curve continues to rise with a decreasing slope, reaching approximately 85 percent recovery at 6,000 articles.

* A light gray shaded area surrounding the line represents the 95 percent confidence interval.

Navigate back to Figure 6.
